# Supplementary material for: Identification of photoperiod-induced specific miRNAs in the adrenal glands of Sunite sheep (Ovis aries)
Source: Front Vet Sci. 2022 Jul 22;9:888207. doi: 10.3389/fvets.2022.888207 (PMC9354845; doi:10.3389/fvets.2022.888207)
Supplement: Supplementary file 1 [file Data_Sheet_1.ZIP › Supplementary file/miRDeep2 file.zip!/miRDeep2 file/Novel_Structure/LP42A3/Novel_254.pdf]

Provisional ID	: NC_019477.2_20372
Score total	: 1.1
Score for star read(s)	: -1.3
Score for read counts	: 0
Score for mfe	: 0.8
Score for randfold	: 1.6
Score for cons. seed	:
Total read count	: 126
Mature read count	: 126
Loop read count	: 0
Star read count	: 0

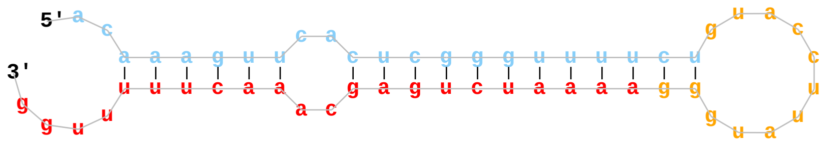

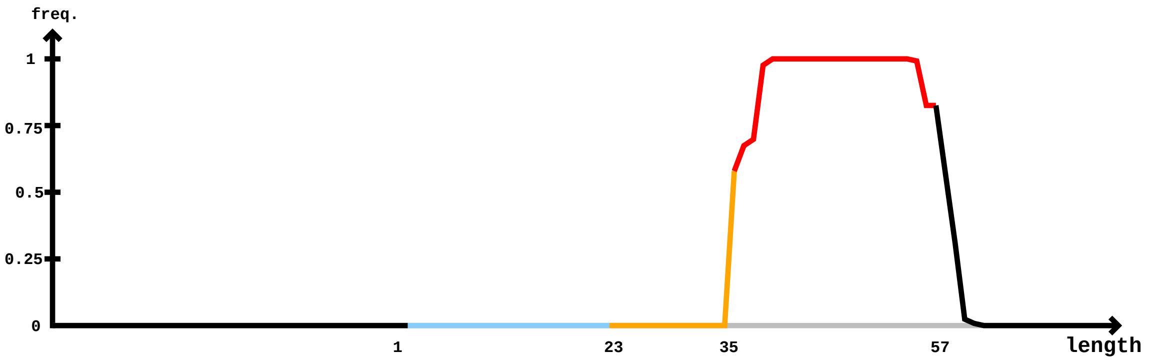

Star Mature

```
5'- uacuugacuuaauuuuuuaauguauucaacuggccacaaaguucacucggguuuucuguaccuuaugggaaaaucugagcaaacuuuuuggccaacccacuauuuucaa   -3'    exp
    ...((((....(((....)))....)))).((((((.((((((..((((((((((((..........))))))))))))..)))))).))))))...............   reads      mm         sample
    .....................................................................aaaaCcugagcaaacuuuu.....................   1          1          seq
    .....................................................................aaaaucugaAcaaacuuuuu....................   20         1          seq
    .....................................................................aaaaucugaAcaaacuuuuugg..................   51         1          seq
    .....................................................................aaaaucugaAcaaacuuuuuggc.................   1          1          seq
    ......................................................................aaaucugagcaaacuuuuu....................   1          0          seq
    ......................................................................aaaucugaAcaaacuuuuugg..................   11         1          seq
    .......................................................................aaucugaAcaaacuuuuuggc.................   1          1          seq
    .......................................................................aaucugaAcaaacuuuuuggcc................   2          1          seq
    ........................................................................aucugaAcaaacuuuuuggcc................   34         1          seq
    ........................................................................aucugaAcaaacuuuuuggccaa..............   1          1          seq
    .........................................................................ucugaAcaaacuuuuuggc.................   1          1          seq
    .........................................................................ucugaAcaaacuuuuuggcca...............   2          1          seq
```
